# Development and validation of a mental hyperactivity questionnaire for the evaluation of chronic stress in higher education

**DOI:** 10.1186/s40359-024-01889-1

**Published:** 2024-07-15

**Authors:** Rubén Fernández-García, Eduardo Melguizo-Ibáñez, Félix Zurita-Ortega, José Luis Ubago-Jiménez

**Affiliations:** 1 Department of Nursing, Physiotherapy and Medicine, University of Almería, La Cañada de San Urbano, Carretera Sacramento s/n, Almería, 04120 Spain; 2 Department of Didactics of Musical, Artistic and Corporal Expression, University of Granada, Granada, 18071 Spain

**Keywords:** Hyperactivity, Stress, University Education, Validation, Instrumental study

## Abstract

**Background:**

Examination and understanding of neural hyperactivity are some of the greatest scientific challenges faced in the present day. For this reason, the present study aimed to examine this phenomenon in the context of higher education.

**Method:**

Likewise, this work will enable an instrument to be created to appropriately and reliably estimate neural hyperactivity associated with chronic stress in university students undertaking a Physiotherapy degree.

**Results:**

Analysis of content validity was carried out according to agreement and consensus between nineteen experts with Education Science or Psychology degrees, via the Delphi method. On the other hand, face validity was established by administering the questionnaire to a sample of 194 university students aged between 18 and 45 years (M = 30.48%; SD = 13.152).

**Conclusion:**

The final self-report measure, denominated mental hyperactivity, was composed of 10 items which showed adequate fit with regards to face and content validity (*α* = 0.775). Confirmatory factor analysis confirmed that the questionnaire was unidimensional.

**Supplementary Information:**

The online version contains supplementary material available at 10.1186/s40359-024-01889-1.

## Introduction

The default mode network (DMN) has formed the basis of research conducted by Marcus Raichle [1]. The prefrontal cortex, anterior cingulate cortex, praecuneus, posterior cingulate, lateral parietal cortex and lateral temporal lobe contribute to the functioning of this network. Regions within the network join together in the posterior cingulate where they extend to the praecuneus, with this having a direct connection to the hippocampus [2].

Paradoxically, this network first started to be examined in detail when it was uncovered that individuals who were not requested to perform any type of attentional task generated brain activity despite not doing anything [3].

It has been confirmed that this network is vital for brain management and organisation [4]. Scientific studies have proven that, only when a perceptive or motor task is performed [1] which requires attentional processes such as reading a book, listening to a conversation, focusing attention on bodily sensations (for instance, via meditation techniques), etc., does RND activity decrease [5, 6]. Nonetheless, when an individual reflects on memories from the past, such as of personal experiences, or elaborates plans for the future, this same network remains constantly activated. This generates a higher metabolic cost than when performing tasks that are attentional in nature [7]. Studies have demonstrated a relationship between levels of activation in this network and Alzheimer’s, schizophrenia, autism, depression, fibromyalgia and attention deficit [1, 8]. There is also evidence with regards to the relationship of default neural network (DNN) with post-traumatic stress disorders [9], trait anxiety [10] and depression [11].

One of the main functions of the brain is to react to physical and psychological stressors as quickly as possible. When the number of stressors is excessive, an increase in the activity or hyperactivity of the DNN can be produced [1] favouring mental rumination processes [12] and physiological responses to stress [13, 14].

There is currently a broad away of psychometric tools available to measure stress and achieve different objectives. In both clinical and research contexts, overall stress ratings are more common [15]. Given the multi-factorial nature of this variable, tests developed in recent years have focused on increasingly specific aspects and niche areas, for example, acute stress [16], post-traumatic stress disorder [17], etc.

Amongst the most important questionnaires focused on the evaluation of stress using a transactional model, the perceived stress scale (PSS) [18] is found. This scale places emphasis on the subjective perceptions of individuals and their emotional response to stressors. A number of versions of the PSS are available, with the original version, composed of 14 items (PSS-14), and the shortened version, composed of 10 items (PSS-10), standing out. Some studies have found that the 10-item version possesses better psychometric properties when compared with the 14-item version, given that the complete version contains 4 items with weak factor loadings [19, 20]. It should be kept in mind that the PSS-10 has been widely used in epidemiological contexts and clinical research [21]. The psychometric properties of both the PSS-14 and the PSS-10 have been studied with samples from different countries, for example, the United States, Spain, Denmark, Turkey, etc.

With regards to the structural validity of the PSS-14, the strongest evidence pertains to a structure made up of two correlated dimensions [22]. One dimension comprises items that relate to perceived stress or the incapacity to manage it, whilst the other dimension groups together items pertaining to coping capacity and resilience when faced with stress.

With regards to the internal consistency of PSS-14 and PS-10, reasonably strong evidence appears to be available. Studies have relied on the Cronbach alpha, reporting values of between 0.74 and 0.91 for the overall scale in its two versions [23, 24]. Some authors have also employed McDonald’s omega coefficient, finding values between 0.68 and 0.80 for PSS-10 [25].

Coping strategies are directly related with the stress they are hoped to combat. In this sense, a number of scales and questionnaires have been used to evaluate this concept. The ways of coping inventory [26] is currently the most commonly used of these tools.

The majority of the questionnaires currently available to measure coping with stress derive directly or indirectly from the ways of coping inventory. Amongst other questionnaires, the coping response inventory for adults (CRI-Adult) [27] and the multidimensional coping inventory (MCI) conceived by Endler and Parker [28] also warrant a mention.

Attention will now be turned to the COPE (multi-factorial coping questionnaire). This tool was developed within a sample of 978 university students attending the University of Miami. Analysis of the tool pointed to 13, with alpha coefficients between 0.45 and 0.85. The questionnaire contains five scales which measure problem-based coping strategies (active coping, planning, suppression of competitive activities, restricted coping, instrumental social support seeking), five scales pertaining to emotion-based coping (emotional support seeking, positive reframing, acceptance, denial and taking refuge in religion) and three scales that evaluate coping responses (focused on the airing of emotions, behavioural disengagement and mental disengagement) [28].

Muller and Spitz [29] validated COPE in a French population. They found the tool to compose 14 scales in total and to have a strong factor structure and good psychometric properties, in addition to significant correlations between active coping and high self-esteem, low perceived stress and low psychological stress.

Another questionnaire related with this topic is provided by Amirkhan [30] who constructed the coping strategies indicator (CSI) in 1990. This concerns a psychometrically stable instrument with high internal consistency and construct validity. It is based on three dimensions of coping, namely, problem solving, support seeking and avoidance. Internal consistency ranges between coefficients equal to 0.92; 0.89 and 0.83 for support seeking, problem solving and avoidance, respectively [31].

Another relevant questionnaire, the coping response inventory (CRI), it based on a transactional model in which coping strategies act as mediating variables of the relationship between serious life crises and its implications for health and wellbeing. The original instrument possesses 8 scales with alpha coefficients that fluctuate between 0.74 and 0.61 in men, and between 0.71 and 0.58 in women. In addition, with regards to convergent validity, correlation coefficients have been produced that range between 0.95 and 0.56 for the guidance and support seeking, and emotional discharge scales, respectively [27].

Kirchner et al. [27] also conducted a study with the aim of analysing the psychometric properties of the Spanish version of the coping response inventory for adults (CRI-Adult). Their outcomes indicated very good psychometric properties. Reported alpha coefficients ranged between 0.52 and 0.70 in men. In the female population, values between 0.50 and 0.66 were obtained.

It is widely recognised that university students live with specific stressors, stress responses and coping strategies [33, 34]. Moreover, authors such as Souto, [35] consider that stress cannot be understood simply in terms of stimulus, but includes the ability to manage the stimulus or its effect and an integration of these phenomena. The Inventario de Situaciones y Respuestas de Ansiedad-ISRA [36] or others designed specifically for the assessment of the university population [33, 34].

Given the lack of scales, questionnaires and tests adapted to the Spanish context with regards to the concept of mental hyperactivity, alongside the fact that those tools that are available have not generally been adapted to the university student population, the elaboration of further instruments is necessary. An instrument is required for use within this highly specific population, which will provide better validity indices than those produced up to the time of writing in university contexts and will produce data for comparison with previous outcomes. Likewise, the aims of the present study are to: (a) Examine content validity through the agreement and consensus of experts, via the Delphi method; (b) Determine the degree of understanding of the instrument by administering it to a sample of university students; (c) Analyse reliability of the developed questionnaire; and (d) Confirm the dimensionality of the construct through confirmatory factor analysis (CFA).

## Materials and methods

### Participants

The use of experts as a strategy for the rating and evaluation of instruments is fairly common in educational research and constitutes the essential and basic aspect of the Delphi method [32, 37], which has been widely used in multiple research studies [38, 39]. In accordance with that proposed by other previous works [39], two groups were established, each responsible for different aspects of the validation of the instrument design. On the one hand, one group was charged with overall coordination, whilst, on the other, the other group formed the expert panel. The first group was made up by members of the present research team. All of these individuals were knowledgeable about the process, were researchers in the field and were highly competent communicators [40]. The second group (the expert panel) was formed in consideration of diverse criteria [41] pertaining to the expert’s link with the topic under study, their professional experience or expertise and personal qualities. Based on the aforementioned, the chosen expert group was formed by university lecturers and researchers of recognised acclaim in the relevant field of knowledge. It is important to indicate that a sufficient number of experts tends to range between 7 and 30 experts. In the present study, 19 experts participated, all of which were university teachers with a PhD and a degree in Primary Education or Psychology. The sample comprised 45.7% women and 54.3% men, with an average of 15.7 years of teaching experience in higher education.

Based on that discussed above, the methodological approach was divided into the following three phases: preliminary, exploratory and final.

In the preliminary phase, the coordinating group sets out the research issue, selects the expert panel (securing the commitment of experts to collaborate), interprets both preliminary and final research outcomes, and makes any adjustments and corrections they consider necessary.

In the exploratory phase, design of the questionnaire was carried out, starting with an experimental adaptation and finishing with the final version. The first version was submitted to a round of analysis and discussion by members of the coordinating group who established the necessary adjustments and corrections according to the most commonly agreed upon qualitative criteria. This version was then validated in a second round by the selected expert panel. The aim of this was to obtain information pertinent to more stable qualitative and quantitative criteria. For this, experts were, first, selected, then they were invited to participate and administered the questionnaire via email. The first page of this email explained the research topic and incorporated a sign-up sheet in which personal data was recorded. The email also explained the aims of the questionnaire and the way in which it should be completed and, finally, included a copy of the questionnaire for its validation. The questionnaire comprised a three-point Likert scale (high, medium and low) which sought to measure the degree of agreement or belonging of an item in relation to a dimension of interest. It also comprised an open question in order to gather qualitative evaluations about the proposed items. Experts were requested to respond within 30 days. During this time-frame, individuals were followed up, completed scales were gathered in and information was analysed by the coordinating group.

In the final phase, results pertaining to the entire validation process for the final version of the questionnaire were synthesised for its later application within 194 university students aged 18 to 45 years (M = 30.48; SD = 13.152), of which 91 (46.9%) were male and 103 (53.1%) were female. The students in this study were from the University of Almeria and the University of Granada. Data collection and analysis was carried out in accordance with the ethical principles established by the Declaration of Helsinki in 1975 and its update carried out in Brazil in 2013 and University of Granada ethics committee 2966/CEIH/2022. Moreover, a research document explaining scientific aims and research topics was drafted, and to request consent for the students’ participation. After obtaining their agreement to participate in the study, the researchers sent them the questionnaire by email.

### Procedure

#### Instrument development

Once the limitations of the available questionnaires and instruments were analysed, a mental hyperactivity questionnaire was developed. This questionnaire met certain requirements proposed in previous work conducted by Ramos et al. [42], including: (a) Conciseness (few items); (b) simplicity (with regards to its application); (c) employ understandable vocabulary which is adapted to the sample characteristics; (d) include short and compulsory questions with closed-format options; (e) be attractive in design and theoretically supported.

#### General procedure for developing the mental hyperactivity questionnaire

The scale was developed and elaborated in accordance with the principles of psychological evaluation instruments proposed by Cronbach [43]. Content was determined following a literature review and in accordance with expert opinion [44] in line with established recommendations. The instrument was to be administered through closed questions with four potential response options.

#### Development of the mental hyperactivity questionnaire

Taking an initial set of items belonging to diverse questionnaires and scales related with mental hyperactivity and other dimensions such as stress as a basis, the coordinating group elaborated a preliminary experimental version, removing some of the items and dimensions that led to mistakes and induced a degree of complexity to overall understanding of the questionnaire.

Each question was posed alongside the following response options: Never, sometimes, often, always. Respondents proceeded to read each item and selected their response based on its appropriateness according to rational criteria. A total of 10 items formed the basis for development of the first version of the questionnaire. Questions were taken from different original scales, with some being copied literally from their original instruments [19–21], others being adapted and others being specifically rewritten for the present topic [19–21]. The questionnaire was developed specifically for this research.

#### Content validity of the instrument

For examination of questionnaire validity, content validity was defined, alongside the degree to which a given test appropriately represented that which it purported to measure [45]. In order to reach optimum levels of content validity, the expert panel approach was employed, whilst, at the same time, a pilot study was conducted to identify understanding within the study sample of interest. Experts were tasked with evaluating the initial information and questions, whilst also providing a general rating for each item. When carrying out this evaluation, experts were asked to consider understanding or appropriateness of redaction.

With regards to the items, a set of statistical indicators were considered. These included discrimination indices and descriptive statistics for each one of the items. With the aim being able to conclude that the data was sufficiently accurate, it was deemed necessary to conduct reliability and validity analyses. The latter of these was performed in order to comply with psychometric requisites and was conducted through examination of the adequateness of Cronbach reliability coefficients and confirmatory factor analysis outcomes [43, 46]. Statistical analysis was performed using the statistical programs SPSS 24.0, FACTOR Analysis 9.3.1 and M-PLUS 7.

#### Examination of instrument comprehension

In order to examine comprehension, a pilot study was conducted in which the questionnaire was administered to 194 university students (5 to 5 min completion time) and the level of understanding was established according to a qualitative prism. Further, questions, doubts and suggestions emanating from questionnaire development and administration were recorded and taken note of.

### Data analysis

For quantitative data, content analysis, examination of basic descriptive statistics and estimation of internal consistency was carried out using the program SPSS 24.0. Exploratory factor analysis (EFA) was performed using FACTOR Analysis 9.3.1, whilst confirmatory factor analysis (CFA) was conducted using M-PLUS 7.

## Results

With regards to content validity outcomes pertaining to the instrument, data obtained using qualitative techniques were examined using content analysis with the aim of producing evidence in relation to the conceptual, cultural and linguistic validity of the mental hyperactivity questionnaire. Qualitative contributions were complemented by the quantitative responses given by experts in relation to each individual item. The integration of both types of information constituted two independent sources and ensured robust examination of instrument appropriateness.

In order to establish the exploratory factor structure, SPSS 24.0 and FACTOR Analysis 9.3.1 were employed. In the first stage of analysis, descriptive values for the study were calculated. In accordance with the steps recommended by experts [47, 48], all items presenting values higher than 2.00 in tests of dispersion (asymmetry and kurtosis) were retained, as can be seen in Table 1.

**Table 1:** Basic descriptive statistics pertaining to the “mental hyperactivity” instrument

	M	DT	V	A	C
L1. Issue/s that you can’t get out of your head and make it difficult to get to sleep	2.14	0.695	0.483	0.554	0.670
L2. Unease	2.27	0.628	0.394	0.489	0.513
L3. Impatience	2.36	0.730	0.532	0.130	-0.209
L4. Difficulty holding attention at the present time	2.11	0.591	0.350	0.272	0.618
L5. Irritability	2.05	0.569	0.324	0.348	1.218
L6. Difficulty managing unmet expectations	2.07	0.742	0.550	0.431	0.119
L7. Insecurity	2.31	0.761	0.579	0.041	-0.398
L8. Feeling of tension/pain in the jaw and/or neck and/or head	2.25	0.841	0.708	0.284	-0.453
L9. Feeling of physical fatigue	2.19	0.741	0.549	0.451	0.197
L10. Unhappiness	1.78	0.665	0.443	0.603	0.644

Note: M, mean; DE, standard deviation; V, variance; A, asymmetry; C, kurtosis

Following this, as can be seen in Table 2, analysis using the program FACTOR Analysis [44] confirmed that all items should be retained for the pilot test. Outcomes from the Bartlett test (371.3 [*df* = 45; *p* = 0.000]) and the Kaiser-Meyer-Olkin test (KMO; 0.811) were examined. This examination was conducted to confirm whether data was taken from populations with equal variance, sampling adequacy and whether fit was good enough for data to be submitted to factor analysis. All outcomes indicated excellent fit for all items. Cronbach alpha was calculated for consideration as part of reliability analysis, producing a value of 0.775 for the overall scale.

**Table 2:** Factor loadings pertaining to the dimensions of “mental hyperactivity”

Variables	F1
V 01	0.582
V 02	0.688
V 03	0.507
V 04	0.356
V 05	0.524
V 06	0.506
V 07	0.456
V 08	0.468
V 09	0.468
V 10	0.547

Once reliability of the items and validity of the instrument, via EFA, had been examined, CFA was confirmed. This being said, the 10 selected questions were grouped according to a theoretical structure made up of a single component, which had been previously corroborated via exploratory analysis. Finally, CFA was used to obtain the factor structure of the instrument. It can be observed that outcomes confirmed the exploratory model to be fully supported by the data. For all index’s, produced outcomes were appropriate to the proposed model, producing a CFI of 0.919 and a TLI of 0.896. Further, the chi-squared value produced was 329.762 with 45 degrees of freedom. Finally, RMSEA was used to evaluate model fit, with a good index of 0.058 being produced. In this way, aforementioned indices confirmed that the proposed model presented acceptable and reasonable outcomes, supporting the hypothesis that this construct is unidimensional.

**Fig. 1:**
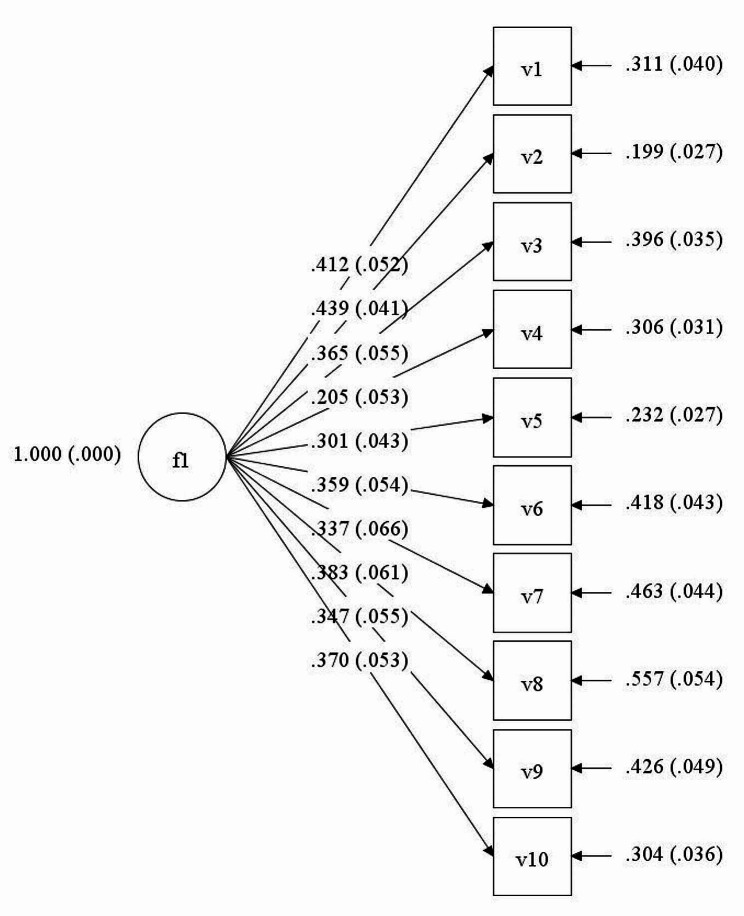
Confirmatory factor analysis of the “mental hyperactivity” instrument

Given these findings, the proposed factor structure was analytically summarised, with estimations of factor saturations for each one of the items within their respective factors being illustrated in Fig. 1.

## Discussion

The aim of the present study was to analyse and validate the content of a mental hyperactivity instrument in a sample of university students undertaking a Physiotherapy degree. It must be indicated that produced outcomes demonstrate satisfactory psychometric quality of the questionnaire following evaluation via confirmatory analysis and show adequate fit to the proposed model.

For the design and validation of the instrument, phases recommended in previous literature were followed [36]. The expert panel that participated in instrument validation (*n* = 19) met quality criteria, with a larger contingent being employed than that seen in other studies with similar characteristics. Specifically, 5 experts participated in the panel recruited by Pereyra-Girardi et al. [49], 6 experts analysed a questionnaire in a study conducted by Hernández-González et al. [50], 10 experts were present in a study carried out by Huéscar and Moreno-Murcia [51], and 3 expert teachers were recruited by Gómez-del-Amo [52]. For this study, most of the experts were from the fields of educational psychology and clinical psychology. The criteria taken into account are set out below: (1) To be simple, feasible and accepted by patients, users and researchers; (2) To be reliable and accurate with error-free measurements; (3) Be appropriate for the problem to be measured; (4) Reflect the theory underlying the phenomenon or concept to be measured and (5) Be capable of measuring changes, both in different individuals and in the response of the same individual over time [42, 43].

The questionnaire also demonstrated sufficient reliability with regards to internal consistency, both in terms of the overall questionnaire and its factor. Obtained psychometric properties pertaining to factor structure and reliability also provide support for the instrument’s content validity and point to good psychometric quality.

Validation of the present instrument, which formed the main aim of the present article, produced evidence to support that it can be considered as an effective tool for indirect evaluation of the state of chronic stress experienced by individuals at determined times in their life. In this sense, the items used to make up the aforementioned instrument provide a direct measure of the potential stressors affecting the general population. To this end, higher scores are representative of increased mental hyperactivity and activation of “rumination” processes [1, 12]. In line with that presented here, scientific evidence confirms that rumination is directly related with stress, depression and alterations to the autonomous nervous system [53]. This generates a physiological response to stress and releases a series of hormones and neurotransmitters which are implied in the development of a number of organic issues [13, 14].

The PSS instrument conceives of stress as implying that events cause the subject to experience a stressor state [18]. This stressor event gives rise to a cognitively mediated emotional response to the target event, not the target event itself [18]. Es por ello que el PSS fue diseñado para medir el grado en el que las personas valoran las diferentes situaciones de su día a día como estresantes [18] The mental hyperactivity questionnaire is not only capable of providing empirical evidence but, also, offers an instrument that can help professionals related with the health field to indirectly determine the degree of DNN activity and chronic stress. Essentially, an interaction exists between the autonomic nervous system (ANS) and the DNN [54, 55]. As discussed previously, exposure to stressful factors over a prolonged period of time leads to excessive “rumination” and alters the DNN and the ANS. As a consequence, the hypothalamic-pituitary-adrenal axis is chronically activated [56–58] leading to chronic stress.

Future research proposals should strive to follow the same line of research taken here in order to replicate the outcomes found in the present study with other samples in other contexts. Main limitations of the present study include the fact that the sample was made up entirely of university students. It should be noted that this sample pertains only to the fields of educational sciences. It could be of interest for future research to employ more heterogenous participants. Another possible limitation pertains to the need to increase the age range of the sample.

## Conclusions

The questionnaire has shown sufficient reliability in terms of internal consistency, both of the overall questionnaire and of its factor. The psychometric properties obtained in relation to factor structure and reliability also support the content validity of the instrument and point to a good psychometric quality.

In conclusion, it is evident that the instrument developed is valid to measure chronic stress during higher education from a one-dimensional perspective.

### Electronic supplementary material

Below is the link to the electronic supplementary material.

Supplementary Material 1

## Data Availability

The data used to support the findings of current study are available from the corresponding author upon request.

## Abbreviations

DMN: Default Mode Network
MCI: Multidimensional Coping Inventory
COPE: Multi-factorial Coping Questionnaire
CRI: Coping Response Inventory
CSI: Coping Strategies Indicator
EFA: Exploratory Factor Analysis
CFA: Confirmatory Factor Analysis
CFI: Comparative Fit Index
TLI: Tucker Lewis Index
ANS: Autonomic Nervous System
PSS: Perceived Stress Scale
DNN: Default Neural Network
