# Supplementary material for: Development and validation of a mental hyperactivity questionnaire for the evaluation of chronic stress in higher education
Source: BMC Psychol. 2024 Jul 15;12:392. doi: 10.1186/s40359-024-01889-1 (PMC11251370; doi:10.1186/s40359-024-01889-1)
Supplement: Supplementary file 1 — Supplementary Material 1 [file 40359_2024_1889_MOESM1_ESM.pdf]

Chronic Mental Hyperactivity Questionnaire

Mental hyperactivity (MH) depends on the "Default Neural Network (DNN)", located in several areas of the brain. This network is activated when we remember the past and make future plans. Its hyperactivity and altered functioning is associated with Alzheimer's disease, schizophrenia, autism, depression, fibromyalgia, attention deficit, post-traumatic stress, anxiety, etc.

To assess the levels of Mental Hiperactivity experienced in the last 3 months, please answer honestly to 10 questions with 4 answer options:

	Never	Sometimes	Frequently	Always
Problem(s) making it difficult to fall asleep				
Unrest				
Impatience				
Difficulty in sustaining attention while performing a task				
Irritability				
Difficulty in managing unmet expectations				
Insecurity				
Feeling of tension/pain in jaw and/or neck and/or head				
Feeling of physical fatigue				
Unhappiness				

The evaluation is carried out using a Likert scale, with the following values:

0= Never

1= Sometimes

2= Frequently

3= Always

References

Akiki, T. J., Averill, C. L., Wrocklage, K. M., Scott, J. C., Averill, L. A., Schweinsburg, B., Alexander-Bloch, A., Martini, B., Southwick, S. M., Krystal, J. H., & Abdallah, C. G. (2018). Default mode network abnormalities in posttraumatic stress disorder: A novel network-restricted topology approach. *NeuroImage*, *176*(May), 489–498. https://doi.org/10.1016/j.neuroimage.2018.05.005

Buckner, R. L., Andrews-Hanna, J. R., & Schacter, D. L. (2008). The brain's default network: Anatomy, function, and relevance to disease. *Annals of the New York Academy of Sciences*, *1124*, 1–38. https://doi.org/10.1196/annals.1440.011

Fernández, R. (2021*). A grandes males grandes remedios: borrón y mente nueva (2ºEd.).* Albacete: Uno

Fox, M. D., & Raichle, M. E. (2007). Spontaneous fluctuations in brain activity observed with functional magnetic resonance imaging. *Nature Reviews Neuroscience*, *8*(9), 700–711. https://doi.org/10.1038/nrn2201

Park, S. M., & Jung, H. Y. (2020). Respiratory sinus arrhythmia biofeedback alters heart rate variability and default mode network connectivity in major depressive disorder: A preliminary study. *International Journal of Psychophysiology*, *158*, 225–237. https://doi.org/10.1016/j.ijpsycho.2020.10.008

Raichle, M. E. (2010). Two views of brain function. *Trends in Cognitive Sciences*, *14*(4), 180–190. https://doi.org/10.1016/j.tics.2010.01.008

Zhang, D., & Raichle, M. E. (2010). Disease and the brain's dark energy. *Nature Reviews Neurology*, *6*(1), 15–28. https://doi.org/10.1038/nrneurol.2009.198

Zidda, F., Andoh, J., Pohlack, S., Winkelmann, T., Dinu-Biringer, R., Cavalli, J., Ruttorf, M., Nees, F., & Flor, H. (2018). Default mode network connectivity of fear- and anxiety-related cue and context conditioning. *NeuroImage*, *165*, 190–199. https://doi.org/10.1016/j.neuroimage.2017.10.02
